# Editorial: Application of multi-omics to important traits of ornamental and beverage plants

**DOI:** 10.3389/fpls.2023.1217267

**Published:** 2023-06-16

**Authors:** Tangchun Zheng, Xue Li, Pervaiz Tariq, Penghui Li

**Affiliations:** ^1^ National Engineering Research Center for Floriculture, School of Landscape Architecture, Beijing Forestry University, Beijing, China; ^2^ Department of Botany and Plant Sciences, University of California Riverside, Riverside, CA, United States; ^3^ State Key Laboratory of Tea Plant Biology and Utilization, College of Tea and Food Science and Technology, Anhui Agricultural University, Hefei, China

**Keywords:** multi-omics, ornamental plant, genetic mechanism, horticultural trait, beverage plant

Ornamental plants contribute to the beautification of the environment, and beverage plants are the raw materials for people’s favorite beverages, both of which play an indispensable role in human production and life. With the rapid development of sequencing technology, omics research has been applied to ornamental plants and beverage plants, resulting in a large amount of omics data, such as genome, transcriptome, proteome, metabolome, etc. However, the study of single omics has great limitations, and there is an urgent need to apply multi-omics to the study of ornamental plants and beverage plants. To explore this interesting research direction, this editorial showcases recent findings and novel insights into “Application of Multi-Omics” to broaden our current understanding of genetic mechanisms responsible for important agronomic, ornamental and resistance traits in ornamental plants and beverage crops.

## Genomes sequencing of ornamental plants

Genome sequencing is an important step toward correlating genotypes with phenotypic characters. Jasmine is famous for its strong fragrance and is used as raw material for scented tea and essence. A draft genome for *Jasmine. sambac* cultivar ‘Danbanmoli’ was assembled by Qi et al., with a size of 520.80Mb and 353,363 predicted genes. Only an ancient whole-genome duplication event was occurred in *J. sambac*, and its divergence from *Osmanthus fragrans* and *Olea europaea* occurred approximately 31.1 million years ago. With the help of multi-omics, numerous transcription factors (TFs) and candidate genes associated with heat stress response and aroma compounds were identified based on the genome, transcriptome and metabolome integration analysis, which lays the foundation for genetic improvement of jasmine flowers.

Another well-known ornamental plant, *Rhododendron*, is used extensively in gardens. Shen et al. assembled a mitochondrial genome of *Rhododendron × pulchrum* with a length of 816,410 bp and 64 mitochondrial genes. 88 simple sequence repeats (SSR) and five genes (*nad1*, *nad2*, *nad4*, *nad7*, and *rps3*) were identified and developed as a molecular marker. The results of genome collinear alignment and phylogenetic tree analyses verified that there was gene rearrangement between *R. × pulchrum* and *R. simsii* mitochondrial genomes, which is useful for the study of population genetics and evolution in Rhododendron and other genera in Ericaceae.

## Multi-omics contribute to color formation mechanism analysis

Cyanidin 3,5-O-diglucoside (Cy3G5G) and peonidin 3,5-O-diglucoside (Pn3G5G) were the primary anthocyanins in rose petals, whose total content and proportion play a role in the depth and hue of the petals, respectively. Wang et al. explored the mechanism of flower color formation in *Rosa rugosa* through a combination of chemical and transcriptome analysis. Thirty-five differentially expressed genes (DEGs) play a role in the upstream pathway of anthocyanin biosynthesis, regulating the total amount of Cy3G5G and Pn3G5G, and *RrAOMT* regulates the ratio of Cy3G5G and Pn3G5G through methylation and determines the color of the petals. Their study provided a novel insight into flower coloration mechanism in *Rosa*.

In *Dendrobium hybrida*, Wang et al. found that *DhMYB2* and *DhbHLH1*, related to anthocyanin biosynthesis, are highly expressed in purple floral tissues. Furthermore, 29 DEGs involved in anthocyanin biosynthesis were screened by transcriptome and their expression patterns are similar to those of *DhMYB2* and *DhbHLH1*. Moreover, yeast one-hybrid (Y1H) and dual‐luciferase reporter assays (DLR) showed that *DhMYB2* and *DhbHLH1* combined with the promoter regions of five structural genes (*DhF3’H1*, *DhF3’5’H2*, *DhDFR*, *DhANS*, and *DhGT4*) to activate their transcription.

To shed light on the regulatory mechanisms of the yellow-leafed *Forsythia*, Zhang et al. measured pigment content and observed leaf anatomical structure of yellow-leaf and green-leaf progenies derived from a hybrid population, and found that yellow-leaf individuals were deficient in chlorophyll and the chloroplast structure. Numerous candidate DEGs (especially *ChlH* and *POLGAMMA2*) were screened out by comparative transcriptome. Moreover, *ChlH* and *POLGAMMA2* changed chlorophyll synthesis and chloroplast ultrastructure by working with several structural genes, which were confirmed by virus-induced gene silencing and transient overexpression analyses.

## Multi-omics contribute to fragrance formation mechanism analysis

Fragrance is one of the most distinctive ornamental characteristics of ornamental plants. Lily is a well-known bulb flower with very high ornamental and commercial value worldwide. To elucidate the mechanism of floral fragrance release, Yang et al. analyzed the transcriptome of 11 stages of flower development, and 4,934 DEGs were enriched in the KEGG pathway and were partially involved in the signaling of plant hormones and monoterpene biosynthesis. Among them, *LiMYB35* was found to be able to activate the *LiOcS* promoter through Y1H, DLR, and EMSA experiments, thus contributing to monoterpenes biosynthesis. These results lay the foundation for understanding the molecular mechanism of lily fragrance.

In another study, Liu et al. identified 1,350 metabolites in *Opisthopappus taihangensis* ‘Taihang Mingzhu’ leaves and inflorescences, of which terpenoids were the most abundant. A total of 82,685 genes were identified in leaves and different stages of flower development by transcriptome, and 43,901 were annotated in protein databases. Additionally, 52 genes involved in the regulation of terpene synthesis were identified by integrating transcriptome and metabolic data. In *Opisthopappus longilobus*,Liu et al. performed transcriptome and metabolomic analysis on leaves, buds, and inflorescences during the exposure, initial opening, and flowering phase. Through GC-MS platform and self-built database, 308 terpene metabolites were detected in leaves, buds, and inflorescences, and 56 candidate genes regulating the synthesis of terpene compounds were identified by transcriptome. These findings may be use to understand the fragrance mechanism of *Opisthopappus* and accelerate the breeding of fragrant flower varieties.

The widely-targeted volatilomics (WTV) and transcriptome were performed by Du et al. to investigate the dynamic changes and formation mechanism of floral fragrance of *Dendrobium chrysotoxum*. Finally, 153 different volatile organic compounds (VOCs) and 4,487 DEGs were identified in flowers of different flowering stages. Furthermore, a transcriptional metabolic regulatory network consisting of four structural genes (*TPSb1*, *TPSb3*, *TPSb4*, and *DXS3*) involved in terpenes synthesis and 100 related TFs was established, which provides a reference for the breeding of fragrant dendrobium.

Tea is the most popular beverage in the world after water. The flowers and leaves of tea plants contain abundant secondary metabolites. Tang et al. explored the metabolic feature of tea flowers and leaves by integrating metabolome and transcriptome. Flowers contain more terpenoid compounds than young leaves, which may be due to higher levels of expression of late-stage genes for the terpenoid biosynthesis pathway in tea flowers. In addition, the distribution of flavonol and catechin was similar in flower and leaves, but key genes involved in flavonoid biosynthesis were selectively expressed by flower and leaves. Their study provides a new insight in mechanisms of biosynthesis and transcriptional regulation of bioactive compounds in tea tree.

## Multi-omics contribute to stress and hormone response mechanism analysis

Cold is one of the key factors affecting plant growth, development and flowering. The sensing and signal transduction mechanism of tea plants to cold stress was summarized by Wang et al. Additionally, the functions and possible regulatory networks of 128 cold stress-responsive genes/families identified by genomics and transcriptome sequencing was systematically summarized through literature review. There was also a discussion of the way in which the application of exogenous treatments to improve the resistance of tea plants to cold stress.

TFs of the three-amino-acid-loop-extension (TALE) gene family play an considerable role in plant hormone regulation pathway and response to abiotic stress. Yang et al. identified 23 TALE genes in *P. mume* genome and found that their promoters contained multiple hormones and abiotic stress response elements. 4 of 11 TALE genes highly expressed in the *P. mume* stem could respond to many hormones and abiotic stresses. Furthermore, fragment replication and tandem replication events were one of the reasons why there were more family members than other homologous species.

To explore the molecular mechanism of *Paeonia ostii* response to waterlogging stress and waterlogging recovery, Zhang et al. performed the full-length transcriptome sequencing of *P. ostii* root by PacBio platform. The study found that *P. ostii* responded to waterlogging stress by adopting hypoxic resting syndrome enhanced waterlogging tolerance by reducing the uptake of nitrate and water from the soil.

*Camellia petelotii* and *C. impressinervis*, both members of the *Camellia* golden subgroup, have different tolerance to high light intensity. Huang et al. discovered that high-light stress caused more severe damage to *C. impressinervis* and a stronger response to reactive oxygen species than *C. petelotii*, possibly due to the reduction of formation and stability of photosynthetic photosystem II and photosystem I complexes and the interconnecting electron transfer chain. Moreover, *C. petelotii* could promote the synthesis and transduction of high trans-zeatin signaling in response to high-light stress and maintain normal growth and development.

Triptolide (TPL), a compound isolated from *Tripterygium wilfordii*, has the effect of treating many human diseases. To decipher the molecular mechanism of TPL toxicity, Luo et al. described the characteristics of protein-coding genes, long-chain non-coding RNA, and cyclic RNA by deep RNA-seq analysis. Finally, c-Jun was screened as a candidate target for TPL and Per1 related circadian rhythm signals were involved in TPL-induced nephrotoxicity.

Li et al. performed a comparative analysis of the genome and transcriptome of *Prunus mume* and *P. persica* to explore the molecular mechanism of early flowering in *Prunus* plants. A total of 19,169 orthogonal groups were identified between the *P. mume* genome and the *P. persica* genome. Genes belonging to the orthogonal group account for 92.4% of the *P. mume* genome and 91.2% of *P. persica*, respectively. In addition, 305 DEGs including three hub genes (*FT*, *TLI65*, and *NAP57*) involved in the early flowering regulation pathway, and 25 TFs were identified, which provides insights in the molecular mechanism of flowering time regulation in *Prunus* genus.

## Multi-omics contribute to crop reproduction and breeding

The difficulty of asexual reproduction is one of the main factors limiting the popularization of peony. Using embryo as explant to induce callus is of great significance to improve peony regeneration system, peony breeding and the industrial development of peony. Zhu et al. selected the best explants and induction medium for the peony ‘Fengdanbai’ embryogenic regeneration technology system. A total of 3,470 DEGs, including 1,767 up-regulated genes and 1,703 down-regulated genes, were identified based on comparative transcriptome analysis of ‘Fengdanbai’ *in vitro* embryos with different proliferation capability. GO and KEGG enrichment analysis showed that DEGs were mainly enriched in the metabolic pathway of phenylpropanoid biosynthesis, which provides a reference for improvement of regeneration technology system of peony.

The unusually long vegetative period is a critical factor limiting the ornamental value of *Cymbidium orchids*. Ahmad et al. found that, under specific cultural conditions, three species of cymbidium could flower within six months by skipping the vegetative period. Hormones and flower regulators are speculated to be the determining factors in this phenomenon. Numerous of DEGs related to hormones, floral integrators and MADS-box genes were screened by transcriptome of leafless flowers and normal vegetative leaves. Moreover, the expression pattern of auxin, gibberellin, and cytokinin relative genes varied greatly among different *Cymbidium* species.

Increasing demand for food from a rapidly growing global population and a changing climate have put enormous pressure on global agricultural systems. Applying genomics, transcriptomics, proteomics and metabolomics to agriculture will facilitate the analysis of the complex genetic properties of food crops, so as to cultivate new crop varieties with positive adaptability to climate change and enhance food production. Recently, Mahmood et al. reviewed the application of the multi-omics revolution in the crop genetic mechanism analysis, and presented profound insights on how to use the multi-omics integration strategy to cultivate crop varieties with desired traits.

In brief, the studies mentioned above can be a foreground to set up the next targets for altered ornamental and beverage plants with desired important traits. Multi-omics analysis is an effective tool to decipher the genetic mechanism of plant key traits and resistance. With the application of omics strategies to plant genetics and breeding research, we are facing greater prospects and challenges.

## Author contributions

TZ and XL drafted the manuscript. All the authors contributed to the article and approved the submitted version.

